# Arthroscopic Management for the Unstable Inferior Leaf of the Lateral Meniscus Anterior Horn and Associated Cysts through a Direct Inframeniscal Portal: A Retrospective Study

**DOI:** 10.1155/2017/9264907

**Published:** 2017-11-13

**Authors:** Dongyang Chen, Qiangqiang Li, Ye Sun, Jianghui Qin, Yao Yao, Qing Jiang

**Affiliations:** ^1^Department of Sports Medicine and Adult Reconstructive Surgery, Nanjing Drum Tower Hospital Affiliated with the Medical School of Nanjing University, Nanjing, Jiangsu 210008, China; ^2^Laboratory for Bone and Joint Diseases, Model Animal Research Center, Nanjing University, Nanjing, Jiangsu 210061, China; ^3^Department of Sports Medicine and Adult Reconstructive Surgery, Nanjing Drum Tower Hospital, Clinical College of Nanjing Medical University, Nanjing, China; ^4^Department of Orthopedics, The First Affiliated Hospital of Soochow University, 188 Shi Zi Road, Suzhou 215006, China

## Abstract

**Introduction:**

To investigate the clinical results of arthroscopic management for the unstable inferior leaf of the lateral meniscus anterior horn and associated cysts through an inframeniscal portal.

**Methods:**

From March 2005 to October 2014, 64 patients with an unstable inferior leaf of the lateral meniscus anterior horn and associated cysts underwent arthroscopic management with an inframeniscal portal. The mean age of the patients was 36.9 years (range, 18 to 49 years). The mean follow-up period was 28 months (range, 24 to 44 months). Clinical results were assessed using physical examination, the Lysholm knee score, and postoperative magnetic resonance scanning.

**Results:**

The median Lysholm score improved significantly at 1 year after surgery and at final follow-up. Magnetic resonance scanning at least one year after the operation revealed no recurrent meniscal tears or cysts. No reoperations were required after an average follow-up of 28 months. All patients reported significant symptomatic relief after the operation. They had full range of motion at three months and returned to normal activities and sports one year after surgery.

**Conclusion:**

The direct inframeniscal portal can provide an effective approach to manage lesions in the anterior horn of the lateral meniscus with predictable clinical outcomes.

## 1. Introduction

In arthroscopic surgery, meniscectomy or meniscal repair is the standard procedure for managing a meniscal tear [1]. This procedure is commonly carried out through the standard anteromedial and anterolateral portals, especially for tears in the midbody and posterior horn. However, arthroscopic surgeons might encounter difficulty when managing tears located in the anterior horn. Isolated anterior horn tears are rarely described in the literature. Shepard et al. [2] reported that the incidence of lesions of the anterior horn of the meniscus was less than 8% by magnetic resonance imaging (MRI) examination and arthroscopic findings. Similarly, Hagino et al. [3] reported a relatively low incidence of isolated anterior horn among a total of 499 cases of meniscus tear. Despite the rarity of an isolated anterior horn tear, the biomechanical studies of the knee showed the torn meniscus could affect the transmission of load, stability, lubrication, and cartilage nutrition [4–6]. Hence, arthroscopic partial meniscectomy has been recommended to be a proper treatment for irreparable meniscal tears [7]. In addition, a meniscus tear, in some cases, could be accompanied with an anterolateral parameniscal cyst [8]. When managing the isolated anterior horn horizontal tear of the lateral meniscus and accompanied anterolateral parameniscal cyst under arthroscopy, the resection of the superior leaf is easy; however, the resection of the inferior leaf, which is more commonly found unstable [9, 10], is much more challenging [11]. By routinely scoping through anterolateral portals and passing instruments through anteromedial portals, visualization of the inferior leaf is poor and the meniscectomy procedure cannot be easily completed [7], resulting in an increased incidence of recurrent tears and iatrogenic injuries. Although visualization of the anterior horn could be improved by passing the scope through the anteromedial portal, the meniscectomy procedure through the anterolateral portal is difficult to be accomplished [12].

Several techniques have been used for the arthroscopic management of anterior horn tears of the lateral meniscus. Kim and Park [7] suggested an arthroscopic technique using three portals but did not present their clinical results in detail. Na et al. [13] developed an extremely far anteromedial portal to improve visualization, but at the same time it has the disadvantage of disturbance of the medial collateral ligament. Kim et al. [11] and Jo et al. [14] developed an inframeniscal portal, whereas both of them did not evaluate the objective postoperative outcome. We developed a direct inframeniscal portal to manage the horizontal tear and cysts located on the anterior horn of the lateral meniscus. The purpose of this study was to introduce a new surgical technique for arthroscopic management of the unstable inferior leaf of the lateral meniscus anterior horn and associated cysts and to investigate the clinical results. Our hypothesis was that this surgical technique would be a simple method that leads to good clinical results.

## 2. Material and Methods

### 2.1. Participants

Between March 2005 and October 2014, one hundred and seventy cases in 170 patients who underwent arthroscopic meniscectomies for an unstable inferior leaf of the lateral meniscus anterior horn and associated cysts were retrospectively investigated. All cases were diagnosed as anterior horn tears of the lateral meniscus preoperatively by MRI. We included patients who met the following criteria: (1) undergoing arthroscopy by a single surgeon; (2) at least two years of follow-up; (3) being examined by MRI of the knee before and at least once after the operation; and (4) having a Lysholm score acquired at three months and each year after the operation. Exclusion criteria were as follows: (1) patients with lower limb malalignment, ligament instability, and severe osteoarthritic changes and (2) patients who underwent other surgical procedures together with meniscectomy, such as ligament reconstruction and osteochondral transfer [15]. Finally, 64 patients met the selection criteria and were included in the present study (Figure 5). Institutional review board approval was obtained before we initiated the study. All patients provided written informed consent.

### 2.2. Surgical Technique

**Figure 1:**
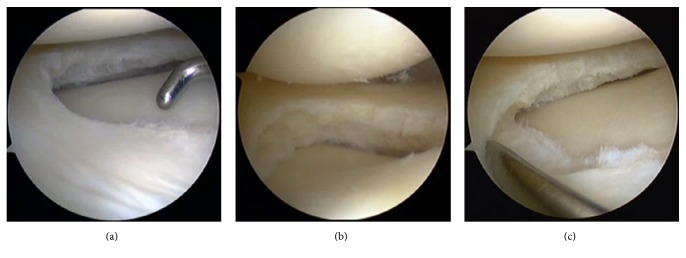
Exposure and examination for the horizontal tear located in the anterior horn of the lateral meniscus. (a) An “intact” meniscus was shown under arthroscopy through the anterolateral portal. (b) A horizontal tear was identified by transferring the scope to the anteromedial portal. (c) A probe passed through the anterolateral portal helped to expose and palpate the tear.

**Figure 2:**
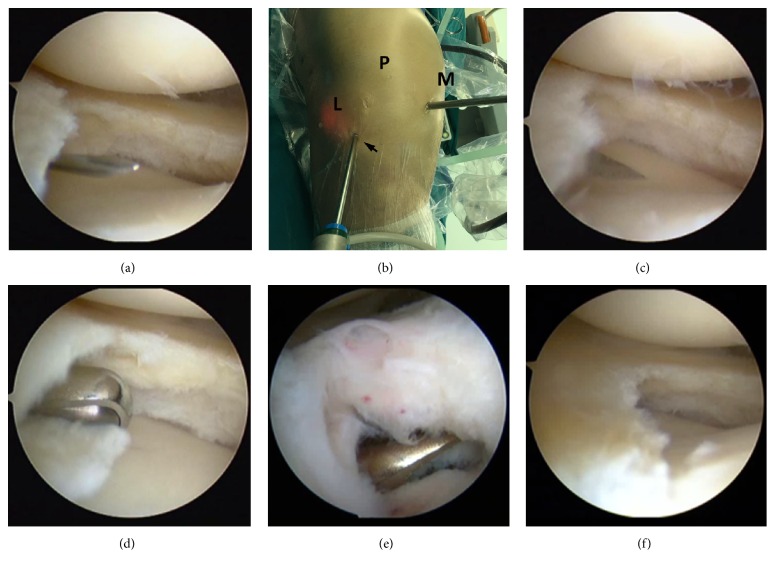
Meniscectomy procedure through the direct inframeniscal portal. (a) A spinal needle was inserted into the interval between the anterior horn and tibial plateau, or into the tear if it extended to the capsule, 1 cm lateral to the patellar tendon. (b), (c) A 5 mm horizontal incision was made by a sharp scalpel (black arrow) and penetrated the capsule just beneath the meniscus or through the tear. (d) The motorized shaver and back-biting cutter passed through the inframeniscal portal to resect the unstable inferior meniscus and then to smooth and contour the remnant rim of the meniscus under monitoring by a scope through the anteromedial portal. (e) Resection of a cyst located in the front of the anterior horn. (f) The anterior horn of the lateral meniscus after meniscectomy. L = lateral, M = medial, and P = patella.

**Figure 3:**
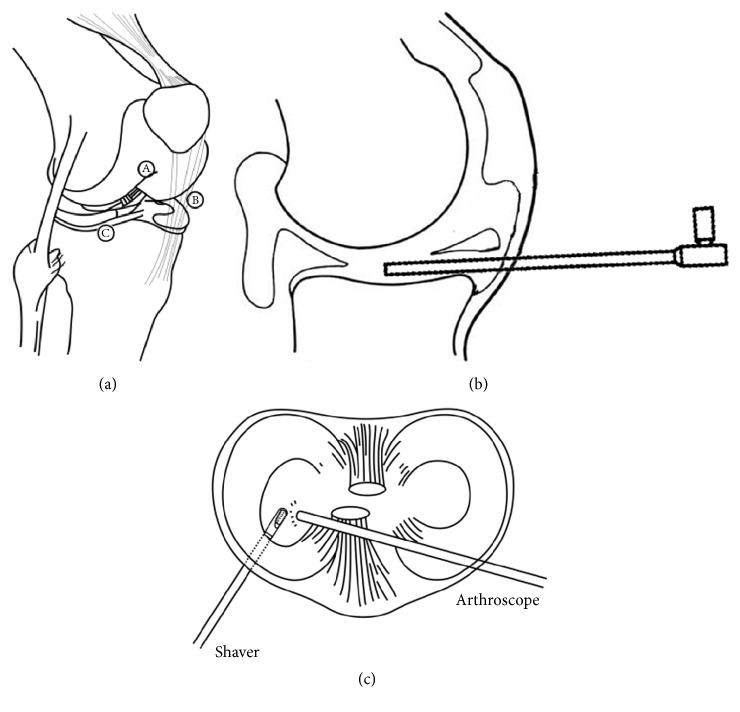
(a) Overall schematic of the inframeniscal technique and three portals. (A) Lateral patellofemoral axillary portal. (B) Medial patellofemoral axillary portal. (C) Inframeniscal portal. (b), (c) Schematic of arthroscopic view illustrates the inframeniscal portal.

A standard knee arthroscopy setting was applied. First, the standard anteromedial and anterolateral portals were made. The joint was inspected routinely; then debridement and meniscectomy were carried out in the posterior portion and middle zone of the lateral meniscus under the figure-of-four position. These procedures were done by direct punch through the anterior medial portal. When gradually moving the punch to the anterior horn, the surgeon would encounter a point where the inferior leaf was not accessible by the punch, and an “intact” meniscus was shown, but the tear could be neglected without careful examination and palpation (Figure 1(a)). By transferring the scope to the anteromedial portal, visualization was improved and the tear could be identified (Figure 1(b)). A probe passing through the anterolateral portal was used to elevate the superior leaf to expose and palpate the horizontal tear (Figure 1(c)). When the range of the tear was determined and a decision for inferior leaf resection was made, a spinal needle was inserted (almost 1 cm lateral to the patellar tendon) into the interval between the anterior horn and tibial plateau or into the tear if it extended to the capsule (Figure 2(a)). After removing the needle, a sharp scalpel was utilized to make a 5 mm horizontal incision (Figure 2(b)) and penetrate the capsule just beneath the meniscus or through the tear (Figure 2(c)). The motorized shaver (4.5 mm Dyonics Synovator blade, Smith & Nephew, MA, USA) that passed through the inframeniscal portal was used to resect the unstable inferior meniscus, followed by smoothing the remnant rim of the meniscus with the shaver, which was monitored with a scope through the anteromedial portal (Figure 2(d)). Schematic outline of the portals is demonstrated in Figure 3.

When a meniscal cyst was concurrently found with the anterior horn tear, the decompression could be carried out through the inframeniscal portal before resecting the inferior leaf of the meniscus. The scope was advanced through the anterolateral portal to visualize the synovium anterior to the root of the meniscus, and a 2.5 mm shaver was brought through the anteromedial portal to make a 1 cm incision on the synovium, which is part of the cyst in most cases (Figure 2(e)). Then, the scope was transferred to the anteromedial portal, and the shaver was passed through the inframeniscal portal to trim the inferior part of the cyst under direct visualization with the scope. The connection between the superior meniscus and the joint capsule was preserved. The resection of the inferior leaf of the meniscus was carried out as described previously.

The stability of the anterior horn was evaluated by palpating with a probe and bringing the knee in flexion and extension (Figure 2(f)). If the anterior horn was stable, the soft tissue defect anterior to the meniscus could be left to heal without suture. If the defect was more than 2 cm after cystectomy and instability of the anterior horn was confirmed, the meniscus and capsule were sutured with polydioxanone (PDS, Ethicon Inc., Somerville, NJ) using an all-inside repair method. Intra-articular fluid was evacuated before the incision was sutured with absorbable sutures. No drainage was used. The knee was wrapped in a dressing with general pressure for five days.

### 2.3. Postoperative Treatment and Rehabilitation

The knee was inspected on the second day. Knee flexion was limited to 90° for the first two weeks, when limited walking was allowed to decrease swelling and facilitate healing between the meniscus and tibial plateau. After two weeks, patients were encouraged to use a full range of motion. They were also encouraged to walk and run to enhance the strength of the quadriceps and to return to normal activities [15]. For patients undergoing all-inside repair of the capsule, walking and partial weight bearing were recommended to start 2 weeks following operation.

### 2.4. Clinical Evaluation

**Figure 4:**
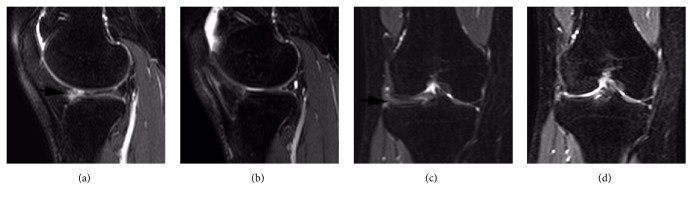
(a), (c) Horizontal tear of the anterior horn of the lateral meniscus was observed in a sagittal and coronal MRI in a 19-year-old college soccer player (black arrow). (b), (d) Sagittal and coronal MRIs one year after the operation revealed the resected inferior leaf of the lateral meniscus anterior horn.

**Figure 5:**
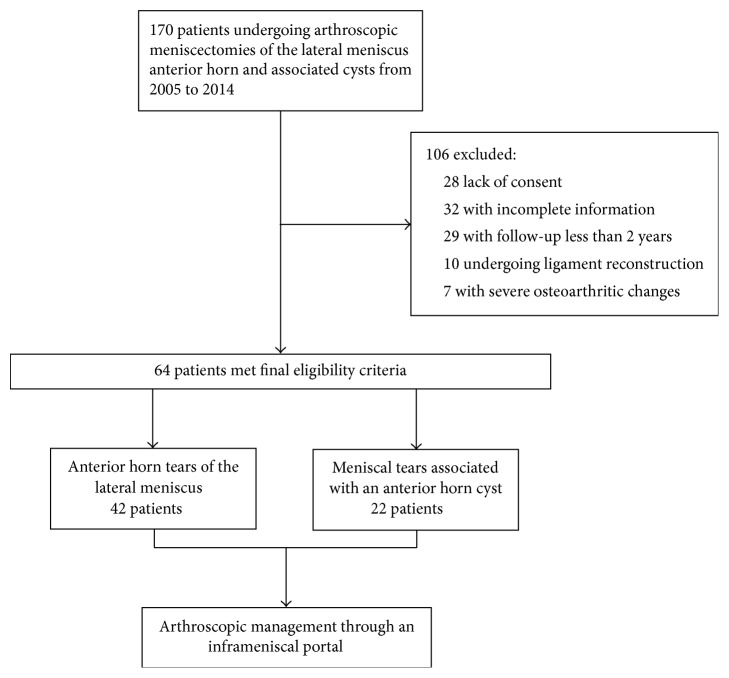
Flow diagram of the study.

Patients were followed up at three months and then each year after the operation. At each visit, clinical results were assessed by the first author. Comprehensive evaluations, including joint-line tenderness, range of motion, joint effusion, and the McMurray test, were checked at a 2-year follow-up. We also evaluated preoperative and postoperative Lysholm scores and range of motion at three months and each year following the operation. T1-weighted, T2-weighted, and proton-density coronal and sagittal view MRIs were used to diagnose the meniscus tears at 3 months and 2 years postoperatively (Figure 4).

### 2.5. Statistical Analysis

Statistical analysis was performed using SPSS 19.0 system software (SPSS Inc., Chicago, IL, USA). Numeric data are shown as the mean ± standard deviation. Continuous variables including Lysholm scores were analyzed by the Student *t*-test and a *P* value of less than 0.05 was considered statistically significant. The sample size was estimated through the following method. The power for the primary endpoint Lysholm score is calculated based on a two sided *t*-test with a significance level of 5%. With a sample size of 32 subjects, the trial will have more than 80% power to detect a difference between the preoperative and postoperative Lysholm score.

## 3. Results

### 3.1. Demographic Characteristics

There were 48 males and 16 females with an average age of 36.9 years (range, 18–49 years). The average duration of symptoms was 11.4 months. Symptoms included knee pain, swelling, and locking sensation. Anterior horn tears of the lateral meniscus were diagnosed using MRI in 42 patients (65.6%) and meniscal tears associated with an anterior horn cyst in 22 patients (34.4%). T2-weighted proton-density images were used to diagnose the lesions (Figure 4). Suture of the anterior meniscus was carried out in only three cases. The mean follow-up period was 28 months (range, 24–44 months).

### 3.2. Clinical Outcome

At three months after the operation, all patients returned to preoperative daily life and work. Six patients reported swelling and tenderness at the surgical site after walking for longer than thirty minutes.

At final follow-up, normal range of motion was shown compared to the contralateral knee, and no positive McMurray tests were triggered by physical examination. No patient reported locking or pain in the surgical site when extending the leg. Two patients reported tenderness after sports, while the other patients reported no symptoms. The Lysholm scores increased significantly from 63.1 ± 5.3 before surgery to 82.5 ± 6.2 at three months after surgery and 91.6 ± 2.3 at 1 year after surgery and reached 96.0 ± 3.4 at final follow-up (Table 1). Fifty-five of 64 received MRI examination at 2-year follow-up and the results revealed no recurrent meniscal tears or cysts (Figure 4). With regard to return to sport, twenty-six patients were engaged in sports preoperatively. And 38 patients returned to sport at different levels following surgery. The sports they played included basketball, running, dancing, and badminton.

## 4. Discussion

The aim of this study was to investigate the clinical outcome of arthroscopic treatment of the unstable inferior leaf of the lateral meniscus anterior horn and associated cysts through an inframeniscal portal. In this study, we found good functional outcomes and low incidence of recurrence and minimal complications can be expected with this portal.

The approach to pathologies in the anterior horn of the lateral meniscus is a challenging technique to the arthroscopic surgeon due to poor visualization. To improve the exposure, it has been suggested to pass the scope through the anteromedial portal instead of the routine anterolateral portal [11–14]. However, meniscectomy of the anterior horn of the lateral meniscus becomes even less possible with this method. The inferior leaf is not reachable through the anteromedial portal. To solve this problem, a third portal has been introduced by several authors [11–14]. Park et al. [12] and Na et al. [13] developed an extremely far anteromedial portal to improve visualization. However, disturbance of the medial collateral ligament and the passage of instruments through this portal were a concern [11]. In contrast, Kim et al. [11] and Jo et al. [14] used an extreme lateral inframeniscal portal, which is located just posterior to the remnant anterior horizontal tear of the lateral meniscus [11] or in the middle one-third of the lateral meniscus [14]. Instruments were inserted through the portal beneath the meniscus to manage the tear. In accordance with Kim et al. [11] we found that most of the anterior horn tears deepened to the capsule. Hence, a direct inframeniscal portal was developed so that the punch and shaver could have easy and direct access to the inferior leaf.

With this approach, the arthroscopic surgeon can approach the anterior horn of the lateral meniscus and the inferior leaf more freely, which can be difficult to adequately address with the standard anteromedial and anterolateral portals. In addition to improved visualization and a simplified procedure, this technique has the advantage of avoiding an additional skin incision. All patients in this study reported significant symptomatic relief, full range of motion, and low incidence of recurrence at the latest follow-up, indicating a satisfactory outcome with this new portal.

In addition, the inframeniscal portal showed substantial advantages when it was used to manage cysts associated with the anterior horn of the lateral meniscus. When resecting a cyst using standard portals, the connection between the meniscus and capsule superior to the cyst should be removed for exposure, requiring subsequent suture of the meniscus and capsule, which is technically demanding [9, 16]. By working through the inframeniscal portal, the connection between the inferior meniscus and tibial plateau can be dissected to expose the cyst and any other concurrent meniscal tears. The superior connection between the peripheral meniscal rim and capsule can be preserved. In addition, with the improved visualization of this direct approach, the risk of iatrogenic damage to the root is reduced. By limiting knee flexion in the early weeks after the operation, the connection between the meniscus and tibial plateau can heal. Suture of the anterior meniscus was carried out in only three cases, and none of our patients reported joint locking after surgery, indicating that the anterior horn was stable. Furthermore, the completeness of cystectomy could be confirmed by arthroscopy through the direct inframeniscal portal. This might be the critical reason for no recurrent cysts in these patients with a minimum two-year follow-up.

One limitation of the present study is that it was a retrospective study without a control group. Additionally, we are not sure whether there is difference in clinical outcomes between our technique and the other techniques reported [11–14].

In conclusion, a direct inframeniscal portal was developed to treat the horizontal tear and cysts in the anterior horn of the lateral meniscus. This portal provides easy and direct access for instruments to the cyst and unstable inferior leaf without an additional incision and minimizes disturbance of the anterior root of the lateral meniscus and the connection between the peripheral meniscal rim and capsule. Good functional outcomes, low incidence of recurrence, and minimal complications are expected with this portal.

## Figures and Tables

**Table 1:** The median Lysholm score improvement in patients operated through the direct inframeniscal portal technique.

Patients (*n* = 64)	Lysholm score (±SD)	*P* value^*∗*^
Preoperative	63.1 ± 5.3	
Three-month follow-up	82.5 ± 6.2	*P* < 0.001
One-year follow-up	91.6 ± 2.3	*P* < 0.001
Final follow-up^¶^	96.0 ± 3.4	*P* < 0.001

^*∗*^Significance is identified as a *P* value under 0.05. ^¶^The median final follow-up is 28 months; SD: standard deviation.

## Abbreviations

MRI: Magnetic resonance imaging
PDS: Polydioxanone.
